# Perception of the Effectiveness of Health-Related Campaigns among the Adult Population: An Analysis of Determinants

**DOI:** 10.3390/ijerph16050791

**Published:** 2019-03-04

**Authors:** Mariusz Duplaga

**Affiliations:** Department of Health Promotion, Institute of Public Health, Faculty of Health Sciences, Jagiellonian University Medical College; 31-008 Kraków; Poland; mariusz.duplaga@uj.edu.pl

**Keywords:** health promotion, health behaviours, social campaigns

## Abstract

Background: Social campaigns focusing on health are commonly used within an attempt to change behavior. To date, there has not been a targeted analysis of societies’ general perception about social campaigns. The aim of this study is to assess citizens’ opinions on the effectiveness of health-related social campaigns. Methods: The data set used in the analysis was obtained from Poland’s nationwide “Social Diagnosis” study. The determinants of public opinion were assessed using a multivariate logistic regression. The independent variables included socio-demographic characteristics, lifestyle, social participation, and the use of digital media. Results: The logistic regression model was developed using 23,593 cases. Opinions about the effectiveness of campaigns depended on all the predictors included in the socio-demographic cluster, smoking, self-declared excessive alcohol consumption, physical activity, the use of mobile phones, and watching TV. A significant impact was found in all but one variable related to social participation. Conclusions: The analysis revealed that opinions about social campaigns present in the media “landscape” are influenced by many factors. Interestingly, persons exhibiting unhealthy behaviors are more resistant to health-related campaigns and surprisingly the need to make use of healthcare resources is not accompanied by an acceptance of the interventions.

## 1. Introduction

Today, social marketing is perceived as a promising strategy that can lead to health improvement through behavioral change. In 1971, Kotler and Zaltman defined social marketing as “an application of marketing to the solution of social and health problems” [1]. Andreasen also emphasized the importance of social change accomplished through social marketing [2]. It is expected that social marketing employs key elements originating from commercial marketing including consumer research, segmentation, targeting, and an appropriate balance of marketing mix attributes [3]. However, the analysis of the secondary evidence on the use of social marketing strategies in relation to health challenges shows that these requirements are not always strictly addressed [4].

Behavior change was indicated as one of key benchmarks enabling identification of the success of social marketing programs [5]. According to this approach, genuine social marketing programs should use behavior change in both the phase of design and evaluation. Behavior change is also perceived as one of the key aims of health promotion interventions. The epidemiological transformation that occurred in the 20th century resulted in new challenges for public health with non-communicable or chronic diseases becoming one of the main problems. According to a World Health Organization (WHO) Report, eight leading risk factors causing deaths in 2004 included: High blood pressure, smoking, elevated glycaemia, insufficient physical activity, overweight and obesity, high cholesterol, risky sexual behavior, and alcohol consumption [6]. These were responsible for 50% of deaths worldwide [6]. Most of these risks are strongly linked to individual behavior. Therefore, the modification of health behaviors is an essential outcome for a successful health promotion campaign, especially in the context of non-communicable diseases. However, despite many theoretical frameworks for modifying health behaviors [7] and the efforts made to develop effective interventions, the area remains a major challenge in public health [8,9]. Some authors stress that interventions focused on changing health behaviors, undertaken in order to reduce alcohol consumption, to prevent obesity or to increase physical activity, have only limited impact on the targeted populations [9,10]. A search for new approaches to influence health-related behaviors results from the apparent disillusionment with the existing strategies.

Social marketing, despite many reservations, is a possible approach. The interest in social marketing is based on the recognition that today, lifestyle and health related behaviors are strongly influenced by the overwhelming influence of consumerism and an exposure to commercial marketing. There is growing evidence that the prevalence of chronic diseases is strongly dependent on the consumption of unhealthy products aggressively marketed by the industry [11]. Social marketing, employing similar techniques to those used for commercial marketing, is perceived as an adequate countermeasure.

Furthermore, social marketing is an attractive option for public health campaigns, as it can be used to address many potential objectives; not only shaping individual health behaviors, but also changing attitudes of policy makers and stakeholders who influence the legal environment for health.

The evidence accumulated so far indicates that social marketing techniques may be effective in many key areas for health promotion. Their feasibility was demonstrated in relation to items such as: Youth obesity [12], increasing physical activity among adults [13], addressing global health issues [14], preventing and controlling sexually transmitted diseases [15], distributing health-related products [16], promoting influenza vaccination [17], healthful eating [18], and cessation of smoking [19]. On the other hand, there are also areas of health promotion in which the effectiveness of social marketing techniques was not confirmed [20].

While social marketing programs are usually expected to assume a more comprehensive approach to behavior change, they are frequently limited to campaigns based only on communication interventions. Campaigns utilizing modern communication channels, which are an inherent element of “the landscape” of traditional and digital media have become a popular strategy for delivering health-related messages to society. There are numerous examples of the robust assessment of social campaigns addressing health-related issues such as smoking [21], however, many social campaigns broadcasted by the mass media are not followed by an appropriate evaluation. While techniques of social marketing may be feasible for many health challenges, their use has rarely been assessed in terms of effectiveness or acceptance by the general public.

The primary aim of this study is the assessment of determinants of opinion about the effectiveness of health-related social campaigns by the public. Social marketing has become an important tool for health promotion and disease prevention in Poland. The increased trend of using social marketing accelerated after Poland joined the European Union in 2004, by the ability to use structural funds designed for social change in new member states. Both public institutions and non-governmental organizations began exploring social marketing techniques as a means to promote change in public behaviors and attitudes.

To clarify the perception of health campaigns in Polish society, the dataset of a nationwide study entitled “Social Diagnosis” was explored and relevant data were extracted [22]. A multivariate logistic regression model was developed to determine factors shaping public opinion about the effectiveness of health-related social campaigns. Potential predictor variables were derived from items asking about socio-demographic features, lifestyle, and health status, the use of television (TV), modern technologies, and social participation.

## 2. Materials and Methods

### 2.1. Overview

The analysis of factors determining public opinion about the effectiveness of health-related social campaigns was carried out from data collected during the wave of the Social Diagnosis study performed in 2011. The “Social Diagnosis” is a panel study carried out every two years on a national sample representative of all Poland [22]. The study is focused primarily on the assessment of objective and subjective quality of life. A two-stage households sampling procedure, based on territorial units and categories of the area of residence was used in the study. Respondents of 16 years and over were asked to fill individual questionnaires in the presence of interviewers employed by the study. The data set from the Social Diagnosis study may be accessed from the website maintained by the research team [23]. The details of the sampling procedure and generation of calibrated weights are specified in the report from the study [24].

### 2.2. Dependent Variable

During the 2011 study, an item asking about the respondent’s opinion of the effectiveness of health-related social campaigns was included in the individual questionnaire (“Do you think that social campaigns and other actions aimed at improving health, e.g., antismoking campaigns, campaigns against drugs and promotion of vaccinations, are effective and change people’s behaviour in Poland?”). A dependent variable used for logistic regression model development was derived from this item. It was coded as follows: The response options “decidedly yes” and “rather yes” were treated as confirmation of conviction that social campaigns are effective (coded as ‘1’), and options “decidedly no”, “rather no”, and “difficult to say” as a lack of conviction about the campaign’s effectiveness (coded as ‘0’).

### 2.3. Independent Variables

Four types of independent variables were used in the multivariate logistic regression model. They included sociodemographic characteristics, variables related to lifestyle and health behaviors, variables related to social participation, and finally, variables reflecting the use of media and information technologies (IT).

### 2.4. Sociodemographic and Economic Characteristics

The cluster of sociodemographic variables consisted of gender, age category, education level, place of residence, marital status, occupational status, and net income. The age of respondent was included as a categorized variable with 6 intervals assumed in the “Social Diagnosis” study: 18–24, 25–34, 35–44, 45–54, 55–64, and 65 years or more. Place of residence also was characterized by 6 values: urban areas with >500,000 inhabitants, 200,000–500,000, 100,000–200,000, 20,000–100,000, <20,000 and rural area. Education level was assigned with four options: primary or lower education (level 1 or lower, as classified by the International Standard Classification of Education (ISCED)) [25], lower secondary (vocational education or middle school; level 2 according to ISCED), upper secondary (level 3 according to ISCED), and post-secondary non-tertiary education or university education (levels 4–8 according to ISCED). Marital status assumed four options: ‘married’, ‘unmarried’, ‘widower/widow’, and ‘divorced or remaining in separation’ (formal or actual). Six categories were used for occupational status: ‘employee’, ‘self-employed or entrepreneur’, ‘retired or on disability pension’, ‘university or school student’, ‘unemployed or occupationally passive’, and ‘not provided’. The initial continuous variable providing information about the level of personal net monthly income (average from three preceding months) was categorized into four categories: ≤1000 Polish zlotys (PLN), >1000 to 2000, >2000 PLN per month, and ‘not provided’.

### 2.5. Use of Health Care Services and Lifestyle

Use of health care services during the last year was a dichotomous variable coded as ‘0’, if the respondent used neither public nor private health care services in the preceding year, and as ‘1’ in cases of at least one episode of use. The variable disability status was derived from the relevant item asking about respondent’s being disabled (either formal status when disability was established by decision of a relevant institution in Poland, or informal, when such a confirmation was not sought).

Respondents were asked if they undertake any form of physical activity or sport (no activity vs. at least one type of physical activity or sport). Items asking about current smoking and excessive alcohol consumption were also dichotomous (‘no’ vs. ‘yes’). Finally, the body mass index (BMI) was calculated from weight and height values provided by respondents in the individual questionnaires and categorized by four intervals: <18.5 (underweight), ≥18.5 to <25.0 (normal weight), ≥25 to <30.0 (overweight), and ≥30.0 (obesity).

### 2.6. Television (TV) and IT Use 

In this group, three variables were included: computer use, mobile phone use and TV viewing. Computer use and mobile phone use were dichotomous variables (‘no’ vs. ‘yes’) The variable related to daily TV viewing assumed four values: Not watching TV, watching TV for less than 1 h, for 1–2 h, and for more than 2 h daily.

### 2.7. Social Participation

There were six variables related to social participation used in the model: The number of friends with whom a respondent meets regularly, involvement in activities for the local community, membership in social organizations, participation in religious practices, participation in the last election before the survey, and the ability to indicate a political option. The variable related to number of friends declared assumed 4 values: not more than 2, 3–4, 5–9, and more than 9 friends. The variables indicating involvement in activities for benefit of the local community (district, village or city, neighborhood) and membership to social organizations were dichotomous (lack of involvement or membership vs. confirming activity). Participation in religious services or meetings was assessed based on the number of events attended per month. A relevant variable could assume three values: ‘0’ for those participating in religious services less than once monthly or not participating; ‘1’ for once monthly and ‘2’ for at least twice monthly. Political activity was determined from the response asking about active participation in local government elections, which took place in 2010 (the last elections taking place before the 2011 Social Diagnosis study). The variable could assume three values: not participating in elections, declaring participation, and response not provided. Finally, an independent variable was derived from the response asking for indication of a political party that is closest to the respondent’s views. It assumed three values; ‘no political sympathy’, ‘difficult to say’, and ‘clear political sympathy’. 

### 2.8. Data Analysis

The analysis was performed with SPSS v.21 for Windows (IBM Corp., Armonk, NY, USA). Only data of respondents of 18 years or over were retrieved for the analysis. The frequencies reported in the paper were provided after exclusion of missing values. The factors that could have impact on the opinion about the effectiveness of health-related social campaigns were included as independent variables in the multivariate logistic regression model. The forward method available in SPSS v.21 package was applied. For each independent variable included in the model, odds ratio (OR), and 95% confidence interval (95% CI) were provided. The logistic regression modelling was preceded with assessment of missing values. As the percentage of missing values was lower than 1% for all but one independent variable; in the case of computer use, it was 1.3%, and it was decided that all records with at least one missing value would be removed from the model. Thus, the multivariate logistic regression model was calculated, after adjusting for standardized weights. The analysis of multicollinearity demonstrated that VIF values for independent variables remained in the interval 1.07–2.07. Therefore, all of them were included in the multivariate logistic regression model.

## 3. Results

### 3.1. Characteristics of the Study Group

Weighted number of respondents was 23.593 persons with women composing 55.92%. The percentage of persons with university education or higher was 19.15%, and those with primary education 18.48%. The number of persons working as employees was 35.29%, self-employed 3.86%, farmers 6.42%, and retired or receiving disability pension 33.29% of all respondents.

Nearly 82% of all respondents used private or public health care services at least once in the preceding year. In the study population 14.26% had disabilities. Only 34.20% declared some type of physical activity, 6.14% reported excessive use of alcohol, and 26.28% smoking. 54.91% of the respondents were overweight or obese. Computer use was confirmed by 53.35% of respondents and the use of mobile phone by 81.62%. Only 2.56% reported that they did not watch TV.

Respondents who had no more than two friends were the most numerous group (32.47%). Only 15.91% confirmed involvement in activities for their local communities and 11.62% reported participation in some type of social organizations. 67.93% of respondents reportedly participated in local government elections in 2010 and 45.17% of them indicated political party views close to their own. Finally, 53.35% of respondents expressed the opinion that health-related social campaigns are effective, while the remaining respondents were not convinced about the effectiveness. Detailed characteristics of the study group are provided in Table 1 (the numbers and frequencies are provided for weighted values).

### 3.2. Predictors of the Opinion about the Effectiveness of Social Campaigns

Multivariate logistic regression modelling was performed for 22 variables deemed to have impact on the opinion of respondents about the effectiveness of health-related social campaigns (Table 2). The model demonstrated adequate goodness-of-fit (Hosmer and Lemeshow test chi^2^ = 14.02, df = 8, *p* = 0.081). However, overall, the predicting value of the model was rather limited (the increase of proper classification from 53.4% to 58.7%; Nagelkerke R^2^ = 0.052).

### 3.3. Sociodemographic Determinants

Men were more skeptical about the effectiveness of social campaigns (OR 0.88, 95% CI = 1.04–1.33) than women. As far as age groups, a significant difference was found only for comparison of the youngest group of respondents (18–24) and those in the category of 25–34 years of age (OR 0.88, 95% CI = 1.04–1.34). There were no significant differences between the referential category (18–24) and older age categories. The respondents who achieved higher levels of education were more prone to believe in the effectiveness of health-related social campaigns. The odds that a person with post-secondary or higher education is convinced about their effectiveness were 1.40 times higher than a person with primary education. Respondents living in urban areas (OR 0.89, 95% CI = 0.72–0.87) and small cities <20,000 inhabitants (OR 0.84, 95% CI = 0.76–0.94) were less prone to express belief in the effectiveness of campaigns than those living in urban areas with >500,000 inhabitants. Persons with net income surpassing 2000 PLN were more often convinced about campaigns effectiveness than persons having a net income not surpassing 1000 PLN (OR 1.11; 95% CI = 1.01–1.23). Finally, married persons demonstrated a higher belief in the effectiveness of campaigns than unmarried (OR 0.88, 95% CI = 0.81–0.96), widows/widowers (OR 0.89, 95% CI = 0.80–0.98), and divorced or in separation (OR 0.86, 95% CI = 0.76–0.97).

### 3.4. Lifestyle and Health Status

The opinion about effectiveness of health-related campaigns did not depend on disability status, the use of health care services during the preceding year or BMI. Persons declaring at least some form of physical activity were more prone to believe in campaign’s effectiveness (OR 1.40, 95% CI = 1.07–1.21). Smokers and persons drinking alcohol excessively less frequently showed belief in campaign’s effectiveness (respectively, OR = 0.88, 95% CI = 0.83–0.94, and OR = 0.84, and 95% CI = 0.76–0.93). 

### 3.5. TV and IT Use

There was no difference between computer users and nonusers (OR 1.00, 95% CI = 0.93–1.08). Mobile phone users were more prone to believe in campaign’s effectiveness (OR 1.17, 95% CI = 1.07–1.28). The odds that person watching TV will be convinced about the effectiveness of social campaigns was at least 1.14 times higher than person not watching TV at all. The highest difference was seen between non-watchers and persons watching TV for 1–2 h daily (OR 1.27, 95% CI = 1.09–1.48).

### 3.6. Social Participation

All but one variable related to social participation had a statistically significant influence on the opinion about the effectiveness of health campaigns. The comparison of persons who either undertake or do not undertake activities for local community did not show a statistically significant difference (*p* = 0.53, OR = 1.08, 95% CI = 1.00–1.17). 

Respondents with a higher number of friends with whom regular meetings or contacts were maintained, more frequently believed in the effectiveness of health campaigns. Results for comparisons between persons with not more than two friends were (OR (95% CI)), for 3–4 friends, 1.14 (1.05–1.23), for 5–9 friends, 1.09 (1.01–1.18), and for more than 9 friends, 1.08 (1.01–1.17). 

Furthermore, the odds that a person who declared membership to at least one social organization were 1.10 (95% CI = 1.01–1.20) times higher when compared with a person who did not belong to such organizations. The conviction about the effectiveness of social campaigns was also higher among respondents who participated in elections of local governments (OR = 1.21, 95% CI = 1.14–1.29) and who could indicate a political party which would be the closest to their views (OR = 1.41; 95% CI = 1.33–1.49). 

Persons attending religious services or meetings once or twice monthly more frequently believed in the effectiveness than those not practicing or practicing less frequently than once monthly (OR and 95% CI, respectively; 1.17, 1.06–1.30, and 1.13, 1.06–1.20).

## 4. Discussion

The analysis performed in this paper was based on the large dataset retrieved from the 2011 wave of nationwide Social Diagnosis study. Among the respondents, about 53% were convinced that health-related campaigns are effective. After exclusion of cases with at least one missing value, a multivariate logistic regression model was developed with 23,593 cases. The independent variables used in the model represented four main groups. They included socio-demographic and economic variables, health care utilization and lifestyle, TV and IT use, and finally, social participation. Only four of the independent variables did not exert any influence on the opinion about effectiveness of health-related social campaigns. The level of influence of independent variables that reached statistical significance was not high. The differences between reference categories and tested categories expressed as OR, rarely reached values below 0.80 or above 1.20. The highest differences were seen for the comparison of persons without political sympathies and those with a clear connection with their closest political party (OR = 1.41), for persons who did not participate and those who participated in the last election (OR = 1.21), for persons with the lowest and the highest level of education (OR = 1.39), or those with upper secondary education (OR = 1.28), and finally for the comparison of persons not watching TV at all with those watching it for 1–2 h daily (OR = 1.27).

The people more frequently convinced of the effectiveness of social campaigns, considering sociodemographic characteristics, are women, the persons who reached a higher education level, those living in larger urban areas, persons employed in private or public organizations, people with the highest income, and those living as married couples.

Utilization of health care services during the previous year and disability status did not have a significant impact on the opinion about such effectiveness. It is surprising that utilization of health care services did not have an impact on the opinion about health campaigns. One might expect that persons having more intensive contact with health care systems would also receive some advice on general health risks and therefore should appreciate the importance of health promotion interventions on a societal level. Persons who smoked or declared excessive alcohol drinking expressed a belief about campaign’s effectiveness less frequently. Such an effect was not seen in case of persons with obesity or who were overweight.

Computer usage also did not predict an opinion about the effectiveness of health campaigns. This finding seems to remain in line with the results of the study reported by Redmond et al. [26]. The analysis of the data from Health Information National Trends Survey carried out in the USA in 2005 and 2007, revealed that the use of print media and interpersonal sources of health information exerted a stronger effect on self-reported health behaviors than the use of other sources, including TV and the Internet.

On the other hand, the model developed in our study showed that persons who watched TV at least one hour daily were more in favor of the effectiveness of health campaigns than those who did not watch TV at all. A similar effect was seen among those who used a mobile phone.

Those who were involved in religious participation, engaged in social organizations, had defined political views and declared participation in voting in local government elections preceding the Social Diagnosis study favored the effectiveness of social campaigns.

Unfortunately, it seems that there is a research gap as to the perception of social campaigns by the general public. It is also obvious that more efforts are directed toward the assessment of the needs of potential target groups for specific campaigns. Therefore, a discussion of the results obtained in this study in comparison to other populations is hardly possible. However, opinion about the effectiveness of health-related social campaigns may be treated as an indicator of attitude to public health measures and readiness to accept such communications on key health problems in the society. Assuming this understanding, one can refer to the effects reported of social participation variables used in our study on health behaviors and related outcomes.

The findings reported by other authors tend to agree with the results of our study. Bender et al. demonstrated that higher social capital was associated with a higher probability of participating in a health check-up [27]. In their study, informal socializing and voter turnout was used as measures of social capital. The effect of informal socializing was maintained after inclusion of neighborhood deprivation in the model, and voting turnout became non-significant. Informal socializing was assessed based on responses to two items asking about frequency of contacts with family members and with friends or acquaintances. Neighborhood (census districts) voter turnout was based on the neighborhood participation in the Danish parliament election [27]. Inequality in voter participation may be related to self-rated health. According to Blakely et al., individuals living in the states (USA) with the highest voting inequality had an odds ratio of fair/poor self-rated health status of 1.43 (95% CI: 1.22–1.68) [28].

The beneficial influence of involvement in religious participation/practices on health outcomes, morbidity, adult mortality risk, and longevity was reported earlier [29,30,31]. This effect is attributed to the impact of religious involvement on lifestyle factors and social behaviors [29,32,33,34]. Some authors also emphasize the importance of social support which can be received in religious communities [29,32,33,34,35]. The impact of involvement in religious practices on the perception of the effectiveness of health-related social campaigns seen in our study may be linked to an acceptant attitude toward society level communications encouraging healthy behaviors related to religious involvement.

Interestingly, people demonstrating harmful health-related behaviors are less prone to participate in social activities, e.g., in elections. In the study of Albright et al., daily smokers were less than half as likely as non-smokers to report having voted in the election [36]. The authors suggested that their findings could indicate that there was a link between risky health behaviors and political mistrust. In our study, smokers were also less likely to believe in the effectiveness of social campaigns. It seems that this mistrust may be extended to other domains. It is also important to note, that, although smokers are a key target group for antismoking campaigns, they may be more resistant to this form of health communication than non-smokers.

The number of close friends is treated as one of the indicators of social support. It is commonly accepted that loneliness leads to unfavorable health outcomes and social support is linked to better health, partly because it prevents loneliness [37,38]. Our study revealed that persons with a higher number of friends were more prone to believe in the effectiveness of social campaigns. It is likely that the protective health effect of social support may to some extent be explained by an increase of an acceptant attitude linked to social interaction by health promotion interventions.

### 4.1. Limitations

The study reported in this paper suffers from several limitations. First, it is a secondary analysis of data collected during a study which was not focused on the analysis of determinants of societal approaches to social campaigns. Second, the selection of the variables used as predictors for logistic regression modelling was based on the assumption that they may have an impact on respondents’ views about the effectiveness of campaigns. The Social Diagnosis study encompassed only one item related to social campaigns and a more thorough analysis of relations between respondents’ views and variables established based on the items available in the individual questionnaire was not possible. Among the most important deficiencies, one should enlist the lack of feedback on understanding and being able to properly identify examples of health-related social campaigns. Therefore, it is not fully clear if the selection of the responses showing the lack of belief in the effectiveness of campaigns or inability to express an opinion originates from problems with understanding the concept of social campaigns or an actual opinion about their effectiveness. This weakness was compensated to some degree by the way the item asking about social campaigns was formulated. Two examples of social campaigns were included in the content of the item when asking about their effectiveness.

Finally, the dataset available from each wave of the Social Diagnosis study is very broad and some potentially relevant items could have been overlooked as to their importance for the opinion about social campaigns.

## 5. Conclusions

The perception of the effectiveness of social campaigns by the general public has rarely been addressed so far. It is also not clear how such perception translates into the effectiveness of campaigns measured with health-related outcomes, e.g., actual change of harmful behaviors.

The analysis performed in this paper indicates that the highest conviction about effectiveness of social campaigns was demonstrated by females, persons with higher education and achieving higher income, remaining in marital relation and living in more urbanized areas. Unhealthy behaviors declared by the respondents (higher alcohol consumption, and tobacco smoking) were related to lower belief in the effectiveness of social campaigns. It is not clear if such attitude precedes the beginning of risky behaviors, or it is developed in association with such behaviors. Nonetheless, it seems that people undertaking risky behaviors could be less prone to accept public communication related to health challenges. On the other hand, respondents presenting healthier lifestyles, as confirmed by involvement in physical activities, showed more positive attitudes towards campaigns.

Interestingly, the factors related to social participation which favor the conviction about social campaigns being effective, are also associated with beneficial health effects. This was seen in both the case of election participation and involvement in religious practices.

The finding that recent utilization of health care services does not influence opinions about the campaigns’ effectiveness is unexpected. One could expect that personal experience of searching for medical help would result in a more open attitude toward health-related communication present in the public domain.

## Figures and Tables

**Table 1:** Characteristics of the study population (weighted values).

Variable	Categories	n	%
Gender	female	13,193	55.92
	male	10,400	44.08
Age category	18–24	2864	12.14
	25–34	3555	15.07
	35–44	3547	15.03
	45–54	4334	18.37
	55–64	4759	20.17
	>64	4534	19.22
Education level	primary education	4359	18.48
	lower secondary education	7470	31.66
	upper secondary education	7245	30.71
	post-secondary non-tertiary education or higher level	4519	19.15
Place of residence	urban >500,000	1788	7.58
	urban >200,000–500,000	2033	8.62
	urban >100,000–200,000	1535	6.51
	urban >20,000–100,000	4381	18.57
	urban ≤20,000	3093	13.11
	rural	10,763	45.62
Occupational status	employee	8325	35.29
	self-employment or entrepreneur	911	3.86
	farmer	1515	6.42
	retired or on disability pension	7853	33.29
	university or school student	1537	6.51
	unemployed	3332	14.12
	not provided	120	0.51
Net income *	≤1000 PLN	5789	24.54
	>1000 to 2000 PLN	8561	36.29
	>2000 PLN	3799	16.10
	not provided	5444	23.07
Marital status	married	14,422	61.13
	unmarried	5530	23.44
	widower/widow	2544	10.78
	divorced or separated	1097	4.65
The use of health care services in preceding 12 months	no use	4297	18.21
	at least one episode of use	19,296	81.79
Disability	no	20,228	85.74
	yes	3365	14.26
Physical activity	no	15,525	65.80
	yes	8068	34.20
Excessive use of alcohol	no	22,144	93.86
	yes	1449	6.14
BMI	<18.50	559	2.37
	18.50–24.99	10,080	42.72
	25.00–29.99	8823	37.40
	>29.99	4131	17.51
Smoking	no	17,392	73.72
	yes	6201	26.28
Number of friends	not more than 2 persons	7660	32.47
	3–4 persons	4205	17.82
	5–9 persons	4874	20.66
	from 10 persons	6293	26.67
Activities for local community	no	19,839	84.09
	yes	3754	15.91
Membership of social organizations	no	20,850	88.37
	yes	2743	11.63
Participation in religious meetings monthly	0	6939	29.41
	1	2056	8.71
	>1	14,598	61.87
Participation in the last election held before the survey	no	6957	29.49
	yes	16,027	67.93
	not provided	609	2.58
Declared political option	no political sympathy	9641	40.86
	difficult to say	3296	13.97
	clear political sympathy	10,656	45.17
Computer use	no	11,005	46.65
	yes	12,588	53.35
Mobile phone use	no	4336	18.38
	yes	19,257	81.62
Daily TV viewing	not watching TV	604	2.56
	<1 h daily	2297	9.74
	1–2 h daily	6937	29.40
	>2 h daily	13,755	58.30
Perception of effectiveness of social campaigns	ineffective or undecided	11,005	46.65
	effective	12,588	53.35

^a^ education categories used in the survey were mapped by the levels distinguished by the International Standard Classification of Education (ISCED) classification from 2011 [25]. * In 2011, according to the Polish National Bank, the 1 USD to PLN exchange rate ranged between 2.9822 and 3.4174 and the mean gross salary in Poland was 3400 PLN.

**Table 2:** Multivariate logistic regression results for opinion about the effectiveness of health-related social campaigns.

Variable	Categories	OR	95%CI	*p*
Gender	female/male	0.88	0.83–0.93	0.000
Age category	18–24			0.000
	25–34	1.18	1.04–1.34	0.009
	35–44	1.15	1.00–1.32	0.055
	45–54	0.99	0.86–1.15	0.935
	55–64	0.90	0.77–1.06	0.199
	>64	0.99	0.82–1.19	0.875
Education level	primary education			0.000
	lower secondary education	1.11	1.02–1.22	0.023
	upper secondary education	1.28	1.16–1.41	0.000
	post-secondary non-tertiary education or higher level	1.39	1.24–1.56	0.000
Place of residence	urban >500,000			0.000
	urban >200,000–500,000	0.96	0.86–1.08	0.523
	urban >100,000–200,000	0.89	0.79–1.01	0.063
	urban >20,000–100,000	0.99	0.89–1.09	0.756
	urban ≤20,000	0.84	0.76–0.94	0.002
	rural	0.79	0.72–0.87	0.000
Occupational status	employee			0.004
	self-employment or entrepreneur	1.03	0.91–1.18	0.618
	farmer	1.18	1.03–1.34	0.016
	retired or on disability pension	1.07	0.96–1.20	0.196
	university or school student	1.16	1.00–1.34	0.057
	unemployed	0.93	0.84–1.02	0.131
	not provided	1.15	0.77–1.72	0.496
Net income	≤1000 PLN			0.015
	>1000 to 2000 PLN	1.05	0.97–1.14	0.197
	>2000 PLN	1.11	1.01–1.23	0.029
	not provided	0.95	0.87–1.04	0.247
Marital status	married			0.001
	unmarried	0.88	0.81–0.96	0.004
	widower/widow	0.89	0.80–0.98	0.023
	divorced or separated	0.86	0.76–0.97	0.018
The use of health care services in preceding 12 months	no use/at least one episode of use	0.97	0.90–1.04	0.339
Disability	no/yes	0.94	0.86–1.03	0.170
Physical activity	no/yes	1.14	1.07–1.21	0.000
Excessive use of alcohol	no/yes	0.84	0.76–0.93	0.001
BMI	18.50–24.99			0.113
	<18.50	0.89	0.75–1.05	0.165
	25.00–29.99	1.00	0.94–1.07	0.888
	>29.99	0.93	0.86–1.01	0.073
Smoking	no/yes	0.88	0.83–0.94	0.000
Number of friends	not more than 2 persons			0.020
	3–4 persons	1.14	1.05–1.23	0.002
	5–9 persons	1.09	1.01–1.17	0.029
	from 10 persons	1.08	1.01–1.17	0.030
	not provided	1.01	0.86–1.20	0.878
Activities for local community	no/yes	1.08	1.00–1.17	0.053
Membership of social organizations	no/yes	1.10	1.01–1.20	0.028
Participation in religious meetings monthly	0			0.000
	1	1.17	1.06–1.30	0.002
	>1	1.13	1.06–1.20	0.000
Participation in the last election held before the survey	no			0.000
	yes	1.21	1.14–1.29	0.000
	not provided	1.07	0.91–1.28	0.398
Declared political option	no political declarations			0.000
	don’t know	1.02	0.95–1.11	0.560
	clear political declaration	1.41	1.33–1.49	0.000
Computer use	no/yes	1.00	0.93–1.08	0.982
Mobile phone	no/yes	1.17	1.07–1.28	0.001
Daily TV viewing	not watching TV			0.004
	<1 h daily	1.14	0.97–1.35	0.109
	1–2 h daily	1.27	1.09–1.48	0.002
	>2 h daily	1.19	1.03–1.39	0.020
Constant		0.53		0.000

BMI: body mass index.
